# 

**Published:** 1892-10-15

**Authors:** 


### CONTENTS.

The Bitter Cry of Motherhood - - - - - 33

Medical History from the Earliest Times. By E. T. Withington, M.A., M.B., Oxon. XXVII—Byzantine Medicine 35

The Hospital Clinic—

Moorfields Ophthalmic Hospital.—The Treatment of Corneal Ulcers - - - - - 41

Paddington Green Hospital for Children.—The Treatment of Diphtheria - - - - - 41

Bristol Royal Infirmary.—The Treatment of Fractures of the Shaft of the Femur - - - - - 42

New Drugs, Appliances, and Things Medical.—Unsweetened Condensed Milk—"Furfelt" Chest Protectors—Thiocamph Disinfectant - - - - - 44

New Books and New Editions.—"Sea Sickness" - - - 44

Passing Topics.—Where are the Donors? - - - - - 34

Diet in Disease. By Mrs. Ernest Hart. III.—Stimulants: Alcohol 36

The Attitude of Great Writers Towards Disease. XIII. —Elizabeth Barrett Browning - - - - - 38

Annotations.—Vivisection at the Church Congress—What to do with Female Drunkards—Lord Tennyson's Kindness to Nurses 39

Notes and News - - - - - 40

The Institutional Workshop—

Hospital Administration. V.—Special Hospitals: Ophthalmic and Miscellaneous - - - - - 45

Within the Hospitals.—Devon and Exeter Hospital. - - 46

Hospital Opinion.—The London Homœopathic Hospital - 47

Around the Hospitals - - - - - 48

The Student of Medicine.—Appointments—Vacancies

### EXTRA SUPPLEMENT.—THE NURSING MIRROR.

En Passant.—Royal National Pension Fund for Nurses—Progress at Preston—Bantry Board and its Fever Nurse—Workhouse Nursing at Pembury—Economy at Macroom—Croydon Nursing Institute—Southmolton and its Nurse Association—Northampton Institute for Trained Nurses— xiii
Lectures for Asylum Attendants. By William Harding, M.B.—V. Food - - - - - xiv
Nursing Homes.—VII. Birmingham Workhouse Infirmary - xiv
Girl-Nurses—Miss Meyrick's Nursing Hostel - - xv
Some Australian Experiences—Presentation - - xvi
Everybody's Opinion.—Victoria Jubilee Institute for Nurses, Edinburgh—Brassey Home, Ventnor - - - - xvii
Caldecote House Convalescent Hospital, Bushey Heath xvii
Nurses' Bookshelf—Minor Appointments - - - - xvii
Off Duty.—"The Result of a Chill" - - - - xviii
Appointments—Wants and Workers - - - - xviii

